# Supplementary material for: Differences in Expression of Selected Interleukins in HIV-Infected Subjects Undergoing Antiretroviral Therapy
Source: Viruses. 2022 May 7;14(5):997. doi: 10.3390/v14050997 (PMC9144358; doi:10.3390/v14050997)
Supplement: Supplementary file 1 [file viruses-14-00997-s001.zip › viruses-1619194-supplementary.pdf]

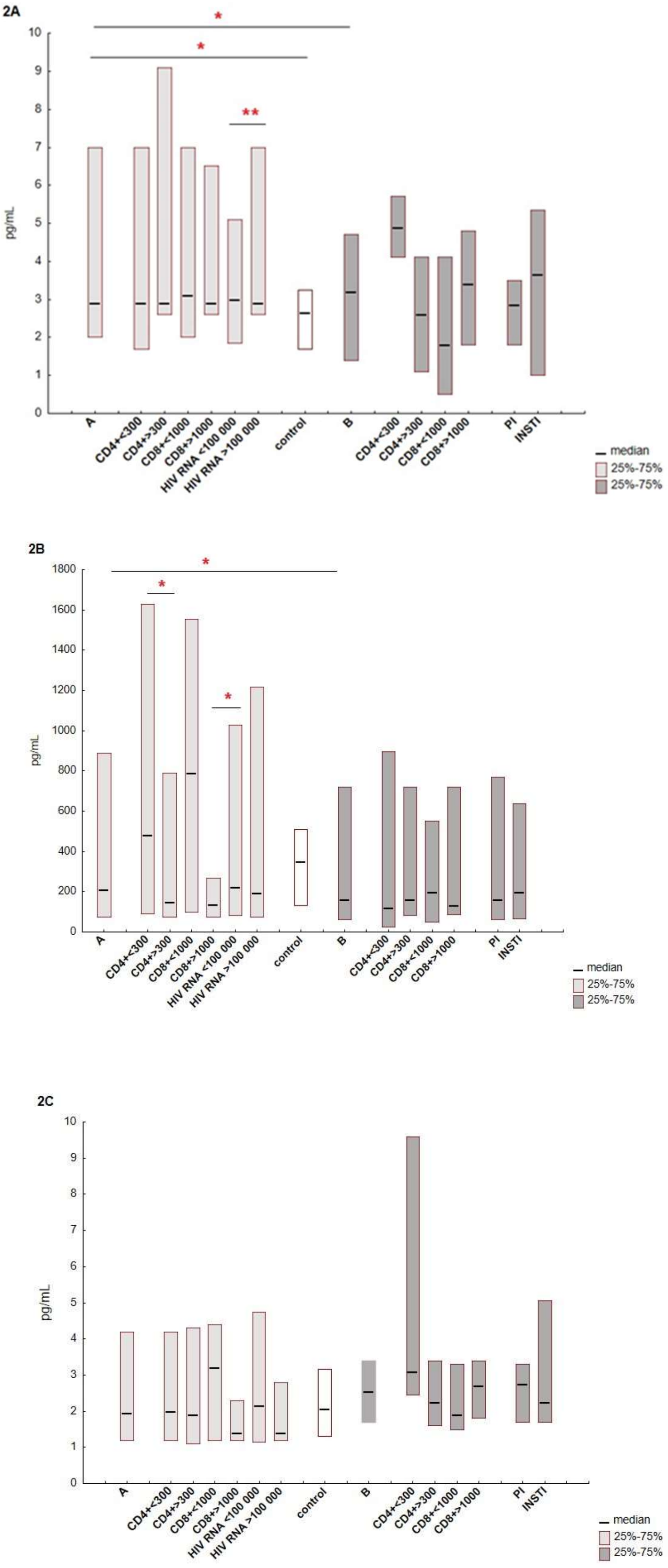

Figure S1. The level of IL-4 (2A), IL-7 (2B) and IL-15 (2C) in blood plasma obtained from HIV-infected men before and after cART depending on the HIV RNA viral load, CD4+ and CD8+ T cell count and on the therapy regimen (PI or INSTI) and control groups; The p-values were calculated using the U Man Whitney and Wilcoxon test; *p<0.05; **p≤0.001
